# What is new in uremic toxicity?

**DOI:** 10.1007/s00467-008-0762-9

**Published:** 2008-08-01

**Authors:** Raymond Vanholder, Steven Van Laecke, Griet Glorieux

**Affiliations:** Nephrology Section, Department of Internal Medicine, University Hospital, OK12, De Pintelaan, 185, 9000 Gent, Belgium

**Keywords:** Convection, High-flux dialysis, Middle molecules, Protein-bound molecules, Removal by dialysis, Uremia, Uremic toxins

## Abstract

Uremic syndrome results from a malfunctioning of various organ systems due to the retention of compounds which, under normal conditions, would be excreted into the urine and/or metabolized by the kidneys. If these compounds are biologically active, they are called uremic toxins. One of the more important toxic effects of such compounds is cardio-vascular damage. A convenient classification based on the physico-chemical characteristics affecting the removal of such compounds by dialysis is: (1) small water-soluble compounds; (2) protein-bound compounds; (3) the larger “middle molecules”. Recent developments include the identification of several newly detected compounds linked to toxicity or the identification of as yet unidentified toxic effects of known compounds: the dinucleotide polyphosphates, structural variants of angiotensin II, interleukin-18,* p*-cresylsulfate and the guanidines. Toxic effects seem to be typically exerted by molecules which are “difficult to remove by dialysis”. Therefore, dialysis strategies have been adapted by applying membranes with larger pore size (high-flux membranes) and/or convection (on-line hemodiafiltration). The results of recent studies suggest that these strategies have better outcomes, thereby clinically corroborating the importance attributed in bench studies to these “difficult to remove” molecules.

## Introduction

A vast proportion of the patients with chronic kidney disease (CKD) suffer from a progressive loss of glomerular filtration, whereby the kidneys lose their capacity to remove potentially toxic compounds from the blood stream into the urine, resulting in their accumulation in the body [1, 2]. As a result of this accumulation, these compounds are called *uremic retention solutes*, and if they are biologically/biochemically active, they are called *uremic toxins*. The accumulation of such compounds has a negative impact on many body functions (Table 1) and results in a gradual, endogenous intoxication. Retention solutes, despite being non-toxic, can still be of interest, as useful markers for other yet toxic elements.

**Table 1:** Organic toxic effects of chronic kidney disease (CKD)

Organic toxic effects of CKD
-Anemia^a^
-Immune dysfunction^a^
-Osteodystrophy^a^
-Hyperparathyroidism^a^
-Insulin resistance^a^
-Malnutrition^a^
-Inflammation^a^
-Coagulatory disorders^a^
-Skin atrophy
-Pruritus
-Polyneuritis
-Coordination disturbances
-Tremor
-Cardiac failure^a^
-Loss of strength
-Anorexia
-Pericarditis^a^
-Hypertension^a^
-Fluid overload^a^
-Cardio-vascular disease^b^

^a^Conditions that conceivably have an indirect or direct impact on cardio-vascular status

^b^Cardio-vascular disease is considered to be the main condition affecting outcome of CKD patients

Among the toxic effects, cardio-vascular damage is a major point of concern because it is responsible for substantial morbidity and mortality, even at the early stages of CKD [3, 4]. Of note, many of the toxic organic effects of uremia (e.g. anemia, inflammation; Table 1) also impact on the cardio-vascular status.

The consideration to classify uremic molecules is inspired by a need to simplify and structure the complex picture of uremic retention, especially within the framework of pursuing more rational, organized and adequate therapeutic approaches. The classification system which is most currently applied at present (see below) is inspired by the behavior of uremic retention solutes during dialysis therapy. It can be assumed that, if new therapeutic and preventive strategies emerge in the future, other classification systems may become appropriate as well. These might be inspired by alternative removal strategies, such as adsorption or convection, by pharmacologic therapies restoring pathways activated or blocked by uremic toxins or by pathophysiological mechanisms per se.

## Current classification

The classification system which currently is most often applied is essentially based on the physico-chemical characteristics of the molecules that influence solute removal by dialysis or related strategies (Table 2) [5].

**Table 2:** Current classification of uremic retention solutes

Classification	Characteristics	Prototypes	Toxicity
Small water-soluble molecules	MW < 500 Da, easily removed by any dialysis strategy	Urea, creatinine	Not necessarily toxic
Middle molecules	MW > 500 Da, removed only through large-pored membranes	β_2_-M, leptin	Large array of biological impacts
Protein-bound molecules	Any MW, difficult to remove with any dialysis strategy	Phenols, indoles	Large array of biological impacts

*MW* Molecular weight, β_2_-M β_2_-microglobulin

Three major groups can be identified:

1. Small water-soluble compounds, whose molecular weight (MW) is arbitrarily defined as 500 Da maximally. The prototypes are urea and creatinine, which are easily removed by any dialysis strategy. Compounds in this group do not necessarily have a marked toxic activity.
2. The middle molecules, whose MW is arbitrarily set at being more than 500 Da. The prototype is β_2_-microglobulin. These molecules can only be removed by dialysis strategies which employ dialyzer membranes containing pores large enough to allow these molecules to cross the membrane (either peritoneal dialysis or high-flux hemodialysis strategies). Shifting the dialysis approach from diffusion, whereby solutes are moved passively from one compartment to another (mostly but not exclusively from plasma water to dialysate) to convection, whereby solutes are actively dragged out of the plasma by imposing water shifts through the membrane, thereby inducing substantial amounts of ultrafiltration (hemofiltration–hemodiafiltration), usually facilitates their removal [6]. Many of the compounds in this group are peptides that affect a host of organ systems.
  Some of the larger middle molecules have a MW of more than 15,000 Da, and they are sometimes called low-MW proteins, although all peptides, even those much smaller than 15,000 Da, semantically and chemically comply with this qualification as low-MW proteins.
3. The protein-bound compounds. Most of the solutes in this group have a low MW, but some have middle-molecule characteristics (e.g. leptin, the cytokines). Prototypes are the phenols and indoles. These compounds are difficult to remove by most of the currently available dialysis strategies, including high-flux dialysis [7]; many of the compounds in this group have toxic activity.

In 2003, the European Uremic Toxin Work Group (EUTox) took the initiative to generate an encyclopedic overview of the uremic retention solutes which were known at that moment, and identified 90 different compounds which were subsequently classified as described above (Table 3) [5]. Since this initiative, several other compounds have been identified, and at least 25 additional solutes have been recognized (Table 3).

**Table 3:** Known uremic retention solutes

Small water-soluble compounds	Protein-bound compounds	Middle molecules
**Listed in the 2003 review by EUTox** [5]		
1-Methyladenosine	2-Methoxyresorcinol	Adrenomedullin
1-Methylguanosine	3-Deoxyglucosone	Atrial natriuretic peptide
1-Methylinosine	CMPF	β_2_-Microglobulin
ADMA	Fructoselysine	β-Endorphin
α-keto-δ-Guanidinovaleriate	Glyoxal	β-Lipotropin
α-*N*-Acetylarginine	Hippuric acid	Cholecystokinin
Arabinitol	Homocysteine	Clara cell protein
Argininic acid	Hydroquinone	Complement factor D
Benzylalcohol	Indole-3-acetate	Cystatin C
β-Guanidinopropionate	Indoxyl sulfate	DIP I
Creatine	Kinurenine	δ-Sleep-inducing peptide
Creatinine	Kinurenic acid	Endothelin
Cytidine	Melatonin	Hyaluronic acid
Dimethylglycine	Methylglyoxal	Interleukin-1β^b^
Erythritol	*N* ^ɛ^-Carboxymethyllysine	Interleukin-6^b^
γ-Guanidinobutyrate	*p*-Cresol^a^	κ-Ig Light chain
Guanidine	Pentosidine	λ-Ig Light chain
Guanidinoacetate	Phenol	Leptin^b^
Guanidinosuccinate	*p*-OHhippurate	Methionine-enkephalin
Hypoxanthine	Putrescine	Neuropeptide Y
Malondialdehyde	Quinolinic acid	Parathyroid hormone
Mannitol	Spermidine	Retinol binding protein^b^
Methylguanidine	Spermine	Tumor necrosis factor-α^b^
Myoinositol		
*N* ^2^,*N* ^2^-Dimethylguanosine		
*N* ^4^-Acetylcytidine		
*N* ^6^-Methyladenosine		
*N* ^6^-Threonylcarbamoyladenosine		
Orotic acid		
Orotidine		
Oxalate		
Phenylacetylglutamine		
Pseudouridine		
SDMA		
Sorbitol		
Taurocyamine		
Threitol		
Thymine		
Uracil		
Urea		
Uric acid		
Uridine		
Xanthine		
Xanthosine		
**Not listed in the 2003 review by EUTox** [5]		
8-OH-2′Deoxyguanosine	Phenylacetic acid	Adiponectin
Dimethylguanosine		Basic fibroblast growth factor
Guanilin		Calcitonin-gene related peptide
Inosine		Desacylghrelin
*N*-Methyl-2-pyridone-5-carboxamide		Dinucleoside polyphosphates^b^
Nitrosodimethylamine		Ghrelin
Nitrosomethylamine		Hepcidin
Phenylethylamine		Interleukin-18^b^
Thiocyanate		Motiline
Trimethylamine		Octopamine
		Orexin A
		Substance P
		Up_4_A^b^
		Uroguanylin
		Vasoactive intestinal peptide

*CMPF* Carboxy-methyl-propyl-furanpropionic acid; *ADMA* Asymmetric dimethylarginine; *DIPI* Degranulation-inhibiting protein I; *SDMA* Symmetric dimethylarginine; *Up*_*4*_*A* Uridine adenosine tetraphosphate

^a^*p*-Cresol, subsequently proven not to be present as such, but as conjugates, such as* p*-cresylsulfate

^b^Middle molecules that are protein-bound at the same time

## Clinical implications

Uremic toxicity affects almost every organ system; therefore, we consider it beyond the scope of this publication to review every toxic effect of each individual compound on all of these organ systems. We have consequently concentrated on cardio-vascular damage with its known major impact on morbidity and mortality [3, 4].

Focusing on the four main cell systems involved in vascular damage necessitates consideration of the leukocytes (monocytes and granulocytes), smooth muscle cells, endothelial cells and thrombocytes. In 2001, the EUTox group summarized the current expertise (Table 4) [2]. With the sole exception of oxalate, all molecules involved in vascular damage were either protein-bound and/or middle molecules, i.e. compounds difficult to remove by standard dialysis strategies. New knowledge collected since 2001 (Table 4) has not changed this perception. It should be noted that one group of compounds included in Table 4, the guanidines, are small and water-soluble, but recent data indicate that their kinetic behavior is nevertheless markedly different from that of our current marker compound, urea [8, 9].

**Table 4:** Uremic toxins with a potential vascular impact

Uremic toxins	Leukocytes	Endothelial cells	Smooth muscle cells	Thrombocytes
**Listed in the 2001 review by EUTox** [2]				
AGE^a^	x^c^	x	x	
AOPP^a^	x	x		
AGE-β_2_-microglobulin	x			
Angiogenin-DIP I	x			
β_2_-microglobulin	x	x	x	
Complement factor D	x			
Cytokines^a^	x	x	x	x
Homocysteine^a^	x	x	x	
Ig-light chain	x			
Leptin^a^	x	x		x
Oxalic acid^b^		x		
**Identified subsequent to the 2001 review by EUTox** [2]				
α-Fibrinogen fragments				x
AII			x	
Guanidines^b^	x			
Indoxyl sulfate		x		
Np_x_N^a^	x		x	
pCS	x			
Phenylacetic acid		x		

*AGE* advanced glycation end products; *AOPP* advanced oxidation protein products; *Np*_*x*_*N* dinucleotide polyphosphates; *AII* structural variants of angiotensin II; *pCS p*-cresylsulfate

^a^Compounds that are protein-bound

^b^Compounds that are small and water-soluble

^c^The "x" indicates that a biological effect was described that interferes with the corresponding cell system and which has the potential to induce in this way vascular damage

The most recently collected information is summarized in the following sections.

### The dinucleoside polyphosphates

Dinucleoside polyphosphates are a group of substances involved in the regulation of vascular tone as well as in the proliferation of vascular smooth muscle cells [10] and mesangial cells [11]. Specific members of this group, the diadenosine polyphosphates, have been detected in hepatocytes, human plasma and platelets. In addition, increased levels of diadenosine polyphosphates are present in platelets from hemodialysis patients [12]. Uridine adenosine tetraphosphate (Up4A) has recently been isolated and identified as a novel endothelium-derived vasoconstrictive factor. Its vasoconstrictive effects, plasma concentration and release upon endothelial stimulation strongly suggest a functional vasoregulatory role [13].

### Structural variants of angiotensin II

Next to genuine angiotensin, structural variants, such as angiotensin A, which is characterized by the decarbonization of the asparagine molecule in the peptide, have been described [14]. These structural variants also have vasoconstrictive properties, be it less prominent than those of genuine angiotensin II. The concentration of these variants is increased in patients with CKD compared to subjects with normal kidney function. It is conceivable that other variants of angiotensin exist, are retained in patients with kidney disease and play a pathophysiological role in vascular dysfunction.

### Advanced glycation end products (AGEs) and advanced oxidation protein products (AOPPs)

Advanced glycation end products and AOPPs are generated by oxidative processes and often incorporated into larger molecules and peptide/protein structures. These, in turn, activate inflammatory processes [15]. Although most of the information obtained from earlier studies was generated by artificially generated AGEs, more recent studies have revealed that also AGE structures present in the body of uremic patients have immune stimulating properties [16]. Recent data suggest that the classical methods for measuring the concentration of AOPPs [17] are biased by an unpredictable background noise created to a large extent by triglycerides and coagulation factors [18, 19].

### Interleukin-18 (IL-18)

Plasma IL-18, a pro-inflammatory cytokine, is increased in patients with CKD, while its concentration is further raised by hemodialysis with bioincompatible membranes [20]. The level of IL-18 is a strong predictor of poor outcome in hemodialysis patients [21].

### *p*-Cresylsulfate

Although most of the pioneering research on the phenolic compounds has focused on the concentration and toxicity of the mother compound* p*-cresol, later work revealed that genuine* p*-cresol is present only at very low concentrations in patients with renal failure and that most of the* p*-cresol generated by the intestinal flora conjugates to* p*-cresylsulfate in the intestinal wall and to* p*-cresylglucuronide in the liver [22, 23]. Both conjugates are characterized by a strong protein binding. The primary reason for the earlier—incorrect—emphasis on* p*-cresol is that most of the earlier determination methods were based on deproteinization by acidification, which causes the disintegration of the conjugates by hydrolysis. Application of deproteinization methods without the acidification step revealed the presence of the conjugate* p*-cresylsulfate [22]. Further studies indicated that the biochemical impact of the mother compound* p*-cresol is not necessarily the same as that of the conjugate. Whereas* p*-cresol suppresses the activity of leukocytes, especially after their activation,* p*-cresylsulfate essentially appears to be linked to baseline leukocyte activation [24]. Nevertheless, since there is very likely a correlation between former* p*-cresol estimations and current* p*-cresylsulfate measurements, previously held conclusions about protein binding and the relationship of * p*-cresylsulfate with clinical outcome parameters of* p*-cresol [25–27] are likely still valid.

### Indoles

The indoles are another group of protein-bound compounds that are generated by chemical transformation processes, such as conjugation. Indoxyl sulfate, the most abundant indolic compound in the body of uremic patients, has been linked to endothelial damage, inhibition of endothelial regeneration and repair, and endothelial free radical production [28, 29]. Indoxyl sulfate has also been related to renal fibrosis and the progression to renal failure [30, 31]. The absorbent AST-120 (Kremezin) decreases serum and urine concentration of indoxyl sulfate in rats [32]. A large prospective clinical study in humans with AST-120 demonstrated a decrease in the plasma concentration of indoxyl sulfate, but showed no clinical benefit [33], possibly because the running time of the study was too short.

### Guanidines

The guanidines are, relative to urea, small water-soluble protein breakdown products. They have long been considered to be neurotoxins [34], but more recently, they have also been linked to vascular damage based on a study demonstrating that several of the solutes of this group activated leukocyte function [35]. In addition, guanidines have also been shown to modify albumin structure in such a way as to decrease the protein binding of homocysteine (HCy) [36], hence stimulating the release of free, active HCy and enhancing the cardio-vascular damaging potential of this compound.

The concentration of another guanidine, asymmetrical dimethyl arginine (ADMA), has been related to several parameters of vascular outcome [37, 38]. Symmetrical dimethyl arginine (SDMA), a structural variant of ADMA, had been considered inert until recently, but it has now been suggested to be related to vascular damage through its inhibition of inducible nitric oxide synthase (iNOS) [39].

Although the guanidines are small and water soluble, they nevertheless show a kinetic behavior that diverges from that of urea, as demonstrated by calculated kinetic analyses [8] and confirmed recently by direct measurements [9]. The distribution volume of most guanidines appears to be much larger than that of urea, hence resulting in more difficult removal and increased rebound at the end of dialysis [8].

## Therapeutic implications

### Diffusive-convective removal through large pore membranes

Since many of the molecules involved in the biochemical/biological side-effects of chronic kidney failure have a relatively high MW, it might be useful to combat those complications by removing those molecules, i.e. dialyzing through membranes with a larger pore size (so-called high-flux membranes). Several studies have convincingly demonstrated that the use of such membranes has the extra bonus of removing several important middle molecules with patho-physiological potential. In contrast, standard dialysis barely removes these compounds or does not remove them at all [40–42]. In fact, the concentrations of these compounds actually tend to increase during standard dialysis due to hemoconcentration as a consequence of the extraction of the extra fluid that has accumulated in the body in between dialysis sessions in combination with no effective removal.

At the end of the previous century, the results of several observational studies suggested that dialysis through large pore (high-flux) membranes resulted in a survival advantage versus dialysis through standard small-pore (low-flux) membranes [41, 43–45]. Controlled prospective studies, however, were not undertaken in this area until 2002, when the HEMO-study was published [46]. The HEMO-study is a randomized trial, undertaken in predominantly American (USA) hemodialysis patients, that comprises four different arms, one comparing standard with high-dose dialysis adequacy, as defined by Kt/V_urea_, and the other comparing low-flux with high-flux membranes. The study protocol adhered to a rather “conservative” definition of high flux with relatively low minimum ultrafiltration coefficients, allowed dialyzer reuse and short dialysis times and was based on a follow-up of 1–5 years. Although this study was unable to demonstrate the superiority of high-flux at the primary analysis with overall mortality as an end-point [46], the secondary analysis revealed that the high-flux membranes had an advantage in terms of cardio-vascular morbidity and mortality [47], cerebrovascular morbidity/mortality [48] and overall mortality in those patients who enrolled in the study after having undergone dialysis prior to enrollment for a longer period than the median time on dialysis of the global group, which was 3.7 years [47]. Another subanalysis showed a relationship between the concentration of the middle molecule β_2_-microglobulin and outcome, irrespective of the type of membrane [49].

Subsequent to the HEMO-study, two studies in dialyzed populations developed with other primary endpoints than the comparison of membrane porosity (one study focusing on nutritional aspects, another on the impact of statins on outcome) demonstrated at secondary analysis a survival advantage for high-flux versus low-flux membranes [50, 51].

The Membrane Permeability Outcome (MPO)-study is a two-armed European controlled study comparing only high-flux and low-flux membranes and essentially recruiting among dialysis patients with a deteriorated clinical condition by focusing on hypoalbuminemic patients [52]. In comparison with the HEMO-study, high-flux was defined more strictly, and dialysis times were longer, reuse was not allowed and follow-up stretched over 3–7.5 years. Among the hypoalbuminemic patients and also in the subgroup affected by diabetes mellitus, a survival advantage was found for patients treated with high-flux membranes (presentation by F. Locatelli at the ERA–EDTA meeting in Barcelona, June 2007).

In summary, many data support the concept that an enhancement of the removal of middle molecules by applying large-pore high-flux membranes creates a survival advantage, especially among dialysis patients with a bleak prognosis, such as the diabetics, the malnourished, the inflamed and those with atheromatosis or at least at risk for atheromatous disease.

Based on information collected before the publication of the MPO-study, the second wave of European Best Practice Guidelines (EBPG) for hemodialysis recommended the use of high-flux membranes [53]. The controlled data collected in the MPO-study has only strengthened this recommendation.

### Convection

If increasing the pore size of the dialyzer creates a survival advantage, adding to the removal of larger molecules by increasing convection may be an additional asset. Several studies have demonstrated that convective strategies enhance dialytic removal of the middle molecules [54–56]. Optimization of this process was previously hampered by the need to substitute ultrafiltered volume by sterile pharmacologically prepared solutions, but the creation of the possibility to generate ultrapure dialysate for substitution purposes has enabled the exchanged volumes to be increased and removal to be maximized.

Data evaluating the outcomes of these convective strategies are relatively scarce. In one prospective study, the prevalence of carpal tunnel syndrome, as a reflection of dialysis-related amyloidosis, was decreased by convective strategies [57]. In the same study, mortality decreased by 10% in the patient group on convection, but this difference was statistically not significant. A prospective comparison of patients on pre-dilution on-line hemofiltration with standard low-flux hemodialysis showed a better quality of life and nutritional status with hemofiltration [58]. In an observational analysis of the DOPPS database, on-line hemodiafiltration with high substitution volumes was linked to a survival advantage [59]. Also, a smaller observational study from the USA showed a striking survival advantage [60]. A Dutch study comparing high-flux hemodiafiltratrion with high-flux dialysis has been started, but the enrolment phase has as yet not been finalized [61].

In conclusion, the suggestion was made as early as 2001, that many of the compounds with the potential to damage the cardio-vascular system have a kinetic behavior that differs from that of urea, the currently universally applied marker of uremic retention and dialysis adequacy [2]. More recent data confirm this position statement: cardio-vascular toxins are quite often uremic retention compounds which are so-called “difficult to remove by dialysis”, either middle molecules, protein-bound molecules or molecules like the guanidines, with a kinetic behavior that differs markedly from that of urea. Given this information, the nephrological community should be aware of the fact that in terms of uremic toxicity, there are more factors at play than urea alone and that when faced with removing uremic toxins, the nephrologist should aim at removing more than only urea.

### Protein-bound molecules

At least three studies suggest a correlation between the concentration of protein-bound uremic retention solutes and clinical outcome parameters [25–27]. However, to date, there are no interventional studies on the effect of improving the removal of protein-bound molecules in uremia on outcome, simply because most of the current removal strategies are not superior to standard hemodialysis in their removal capacity of protein-bound molecules. High-flux hemodialysis has no impact on the concentration of protein-bound uremic retention solutes when compared to low-flux hemodialysis [7]. It is likely that more complex strategies, such as extreme convective therapies or adsorption, will be necessary to induce the removal of any significance.

### Pharmacological interventions

Further study is needed to characterize the as yet unidentified uremic retention solutes and to fine-tune our knowledge of the molecular role of known and newly identified compounds. Instead of concentrating on compounds with a known effect, it may be more rewarding to apply unbiased techniques in the proteomic, genomic and metabonomic fields using refined biostatistic tools to link mediators to clinical outcome. This would appear to be the only approach to identify new elements and mechanisms, which in their turn, in a second stage, may be relevant for the general population as well.

There is also a need for a classification system based on importance of the identified culprits.

In addition to more specific removal methods than the ones applied today, a search for pharmaceutical strategies blocking the molecular impact of uremic solutes is one of the next steps. Such strategies would be based on identified patho-physiological mechanisms and would allow the prevention and treatment in the less severely affected CKD groups long before the need for extracorporeal renal replacement. The final aim here should be to prevent cardio-vascular and other complications and the evolution towards renal replacement therapy.

## Variability in reported concentrations

An often overlooked cause of bias in the interpretation of uremic toxicity is the marked variability in the reported concentrations of uremic retention solutes [62, 63]. Although such a variability has been observed for many solutes, it seems to be a problem that is more frequently associated to the protein-bound molecules, middle molecules and guandines (Table 5). This variability may be at the origin of several potential errors (Table 6), such as the choice of unreliable concentrations of uremic solutes for in vitro experiments, but it may also cause erroneous therapeutic decisions or incorrect interpretation of presumed biological/toxic effects (Table 6).

**Table 5:** Examples of variability in reported concentrations of uremic retention solutes—compounds with the most extreme variability (Data from [62])

	Molecular weight	H	L	H/L
Pentosidine^a^ (mg/L)	342	896	0.2	4112
TNF-α^a,b^ (ng/L)	26000	1419	4.9	289.6
Interleukin-1β^a,b^ (ng/L)	32000	428	2.6	164.6
Cholecystokinin^b^ (ng/L)	3866	526	6.7	78.5
Taurocyamine^c^ (μg/L)	174	6000	78.3	76.6
γ-Guanidinobutyrate^c^ (μg/L)	145	400	10.1	39.6
Neuropeptide Y^b^ (ng/L)	4272	862.9	25.2	34.2
3-Deoxyglucosone^a^ (μg/L)	162	1700	59	28.8

*H* Highest reported concentration; *L* lowest reported concentration; *H/L* ratio of highest over lowest concentration; *TNF* tumor necrosis factor

^a^Protein-bound

^b^Middle molecule

^c^Guanidino compound

**Table 6:** Examples of potential consequences when concentrations are incorrectly interpreted (data from [63])

Compound	Consequences
All	Irrelevant concentrations applied in in vitro experiments
Creatinine	Miscalculation GFR
	Incorrect therapeutic decisions
PTH	Incorrect therapeutic decisions
β_2_-Microglobulin	Incorrect interpretation adequacy of dialysis
ADMA	Incorrect interpretation of capacity to inhibit iNOS

*GFR* Glomerular filtration rate; *PTH* parathyroid hormone; *ADMA* asymmetric dimethylarginine; *iNOS* inducible nitric oxide synthase

## Pediatry

Data on uremic toxin concentration in children are very scanty, and even an intensive literature search results in the finding of extremely scarce data. In one study, the distribution of urea concentrations in relation to kidney function for Italian children was reported [64], there was no consideration of the potential patho-physiological consequences. A better knowledge of the behavior of uremic toxins in children is desirable in view of their differences in metabolism and distribution volume versus those of adults. Hence, it is not acceptable to automatically extrapolate data from the adult population to children.

## Conclusions

In view of dialysis remaining the main therapeutic strategy to counteract the clinical impact of uremic retention molecules, especially at a later stage of CKD, the most practical approach still appears to be that of adhering to a subdivision of uremic solutes into: (1) small water-soluble compounds; (2) middle molecules; (3) protein-bound solutes.

Uremic toxin retention is a complex condition, implying a large number of retention products and far more compounds than only the small water-soluble solutes. Hence, we should be aware of this when interpreting the value of urea or creatinine as markers of uremic retention and realize that there are more players in uremia than urea and creatinine alone. Likewise, removal strategies should be aimed at the removal of more than urea alone, especially since the larger middle molecules and the protein-bound uremic compounds seem to be related to deleterious biological, biochemical and clinical effects. New moieties with a potential impact, especially in the context of cardio-vascular morbidity and mortality, which remain among the main clinical problems in kidney failure, are detected continually and should lead to new therapeutic approaches. The number of larger and/or protein-bound molecules among these compounds also is substantial.

The removal of larger “middle” molecules is likely to be related to better outcomes, as previously suggested by the results of observational studies and recently confirmed in sub-analyses and primary analyses of well-designed controlled studies.

The concentration of protein-bound uremic solutes is also related to clinical outcome but, until recently, no strategies had been characterized that enhance their removal convincingly. With the development of such strategies, it may be expected that it will become possible to design controlled interventional studies evaluating the effect of removing these protein-bound molecules.

Variability in reported concentrations may result in the incorrect interpretation of presumed toxic effects and/or in clinical mistakes.
